# Detection of renal tubular transporter proteins in canine urinary extracellular vesicles using liquid chromatography–tandem mass spectrometry

**DOI:** 10.1111/vcp.13393

**Published:** 2024-10-21

**Authors:** L. J. McGravey, P. R. Antrobus, T. L. Williams

**Affiliations:** ^1^ Department of Veterinary Medicine University of Cambridge Cambridge UK; ^2^ Cambridge Institute for Medical Research, University of Cambridge Cambridge UK

**Keywords:** biomarkers, dog, exosomes, proteomics

## Abstract

Urinary extracellular vesicles (UEVs) are membranous particles that carry renal tubular transporter proteins. Here, we evaluate whether selected renal tubular transporter proteins can be detected in UEVs isolated from small volume (1–5 mL) canine urine samples of healthy dogs and canine patients with elevated circulating parathyroid hormone (PTH)/PTH‐related peptide (PTHrp) concentrations, hypercortisolism, and primary hypoadrenocorticism using liquid chromatography–tandem mass spectrometry (LC–MS/MS). The total creatinine content of each urine sample was calculated from urine volume and creatinine concentration. UEVs were isolated by size exclusion chromatography prior to quantification by nanoparticle tracking analysis and proteomic analysis by LC–MS/MS. Group comparisons were made using non‐parametric statistics. Aquaporin‐2 (AQP2) and the renal sodium/phosphate co‐transporter (NPT2A) were detected in UEVs isolated from small volume samples of almost all healthy dogs but were not detected in most dogs with elevated circulating PTH/PTH related peptide (PTHrp) concentrations, hypercortisolism and primary hypoadrenocorticism. Total creatinine content of the urine sample was strongly positively correlated with the number of UEVs (*r*_s_ = .84, *P* < .001); thus, total creatinine was used as a surrogate marker of UEV number. In healthy dogs, AQP2 and NPT2A were both detected in samples containing at least 1.7 × 10^9^ UEVs or 24 μmol creatinine, however in non‐healthy dogs, AQP2 and NPT2A were not detected in most samples containing up to 6.3 × 10^9^ UEVs or 32 μmol creatinine.

## 1 INTRODUCTION

Extracellular vesicles (EVs) are membrane‐bound particles (50–200 nm in size) produced by all cells that contain proteins from the cells of origin, including renal tubular transporter proteins present in the apical membrane. Urine mostly contains EVs produced by cells from the kidney and urinary tract.^1^, ^2^ Excretion of aquaporin‐2 (AQP2) within urinary EVs (UEVs) correlates with AQP2 expression in the kidney of humans and rats^3^, ^4^ and UEVs isolated from human patients with Gitelman Syndrome (an inherited tubulopathy characterized by reduced renal thiazide sensitive sodium chloride co‐transporter [NCC] expression) contain reduced quantities of NCC compared with healthy volunteers.^5^, ^6^ These data suggest that the UEV proteome is a surrogate marker for the expression of apical proteins within the nephron; however, to date, no studies have investigated whether the proteome of canine UEVs also reflects that of the canine kidney. If the proteome of UEVs was reflective of the renal proteome in dogs, this would support the use of UEVs as a “liquid biopsy” of the canine kidney. The proteome of UEVs could also be used to study the changes in the renal proteome that occur in response to renal disease and endocrinopathies, such as hypercortisolism, in a non‐invasive manner.

The aim of this study was to show proof of concept that renal tubular transporter proteins could be detected within canine UEVs obtained from small‐volume urine samples. We chose to study the EV expression of selected renal tubular transporter proteins that demonstrate altered expression in the renal tubular epithelial cells in naturally occurring canine diseases. The proteins of interest were aquaporin 2 (AQP2), the epithelial sodium channel (ENaC), the luminal potassium channel (ROMK), and the luminal sodium‐phosphate cotransporter (NPT2A or SLC34A1). We hypothesized that UEVs from dogs with hypercortisolism and hypercalcemia would contain reduced quantities of AQP2 compared to control dogs because glucocorticoids inhibit antidiuretic hormone (ADH) secretion^7^ and hypercalcemia inhibits ADH activity,^8^ both of which will lead to a reduction in the mobilization of AQP2 from internal vesicles to the apical membrane of renal collecting duct cells. We also hypothesized that UEVs from dogs with primary hyperparathyroidism and hypercalcemia of malignancy (due to increased serum PTHrP concentrations) would contain reduced normalized quantities of NPT2A because PTH stimulates endocytosis of luminal sodium‐phosphate cotransporter.^9^ Finally, we hypothesized that UEVs from dogs with primary hypoadrenocorticism might contain reduced quantities of ENaC and ROMK compared to control dogs because some studies have suggested that aldosterone influences ENaC and ROMK abundance in renal tubular epithelial cells.^10^, ^11^

## 2 MATERIALS AND METHODS

The study was approved by the Ethics and Welfare Committee of our institution and informed consent was obtained from owners prior to sampling.

Free‐catch urine samples were collected from healthy dogs owned by staff members, and cystocentesis samples were collected from clinical patients with hypercalcemia secondary to primary hyperparathyroidism (elevated parathyroid hormone [PTH] concentrations or increased PTH‐related peptide [PTHrP] concentrations), hypercortisolism, or primary hypoadrenocorticism, as part of clinical investigations. Urine samples were centrifuged at 365*g* for 10 min (within 1–2 h) to separate cellular components, and the supernatant was harvested and stored at −80°C (within 6 h) prior to isolation of UEVs. Samples with evidence of bacteriuria, pyuria or hematuria on microscopic examination were excluded from the study.

Urine samples (1–5 mL) were thawed and vortexed for 30 s prior to analysis. The volume of each sample was measured, and 20 μL of 50× protease inhibitor solution (Pierce Protease Inhibitor, Thermo Fisher) was then added per 1 mL of the sample. The sample was then filtered (0.22 μm syringe filter), and EV fractions were collected as previously published protocols.^12^, ^13^ In brief, the samples were concentrated to ~100 μL volume by centrifugal ultrafiltration (Amicon Ultra centrifugal filter 100 kDa), and then this volume was inserted into a size exclusion chromatography (SEC) column (IZON qEV single 70 nm) and flushed with 5 mL of sterile phosphate‐buffered saline (PBS, Sigma Dulbecco's PBS D8662). The first 1 mL of output from the SEC column was discarded, and the next 600 μL were collected as the EV‐enriched fraction for further analysis.

Nanoparticle tracking analysis (NTA) was performed on the EV‐enriched fraction using Nanosight NS300 and NTA 3.2 software to quantify the size and concentration of EVs. Each EV preparation was diluted to obtain a particle concentration between 5 × 10^7^ and 9 × 10^8^/mL, and 500 μL of diluted EV preparation was injected into the sample chamber at a constant flow rate using the Malvern NanoSight syringe pump system. For all recordings, the camera level was adjusted to 15, and three 60‐s videos were recorded for each sample. For analysis, the detection threshold was set to 5.

Transmission electron microscopy was also performed on the EV‐enriched fraction of a single healthy dog to confirm the presence of particles with the expected morphology of EVs. Glow‐charged electron microscopy grids were placed on a 5 μL droplet of the EV‐enriched sample for 60 s. The grids were washed by placing the grid on two droplets of distilled water for 30 s each; the grids were dried using blotting paper and then counter‐stained by placing the grid on a droplet of uranyl acetate (1.5%) for 45 s. The grids were dried using blotting paper and then imaged using the Tecnai G2 transmission electron microscope.

Samples were reduced and alkylated, followed by tryptic digestion using the S‐Trap spin column platform (ProtiFi, New York). The resulting peptides were analyzed by LC–MS/MS using an Orbitrap Fusion Lumos mass spectrometer configured with an EASYspray source and coupled to an RSLC3000 nanoflow ultra‐high‐performance liquid chromatographic system (nanoUPLC) LC. The nanoUPLC was operated with solvent A (0.1% formic acid) and solvent B (80% acetonitrile, 0.1% formic acid) with peptides resolved on a 50 cm C18 PepMap column using a gradient rising from 7% to 37% solvent B by 88 min at a flow rate of 300 nL/min with the column maintained at 40°C. Spectra were acquired between m/z 350 and 1500 at a resolution of 120 000 full width at half maximum peak height (fwhm) with fragment MSMS generated by higher‐energy collision dissociation (HCD) fragmentation at 34% collision energy. Fragment spectra were recorded in the ion trap at a rapid scan rate with a 2‐s cycle time.

Data was processed in MaxQuant 2.1.0.0 using a Uniprot *Canis familiaris* database (downloaded 11/10/22) with label free quantitation and iBAQ enabled. Analysis of the LC–MS/MS data performed using MaxQuant includes a contaminants database which covers common contaminants found in proteomic samples (keratin, BSA, trypsin, etc.) but not entire bacterial proteomes. Searches were performed using a peptide/spectrum match and protein FDR of 1%, which, when combined with careful manual inspection of peptides from key identified proteins, minimizes the likelihood of peptide misidentification.

The proteome generated from the EV samples was interrogated using Gene Ontology (GO) enrichment analysis (https://geneontology.org/) and correlating the proteome with “cellular component” and the *Canis lupus familiaris* database, to confirm enrichment for proteins from EVs.

The UEV concentration per mL (of urine) was calculated for each sample and indexed to urine creatinine concentration to calculate the UEV: creatinine ratio and provide an indication of urine EV excretion rate. Data were compared between groups using the Kruskal–Wallis test with post hoc Mann–Whitney *U* test or the Mann–Whitney *U* test (as appropriate), and correlations were assessed using Spearman's correlation coefficient, with statistical significance defined as *P* < .05. Data are presented as median [minimum‐maximum].

## 3 RESULTS

The study included six healthy control dogs, five dogs with hypercalcemia secondary to increased PTH/PTHrP activity (3 with primary hyperparathyroidism [PHPT] and 2 with anal sac adenocarcinoma [ASAC] and thus presumed elevated circulating PTHrP concentrations), 6 dogs with hypercortisolism and 3 dogs with primary hypoadrenocorticism. Data on breed, age, and sex of the dogs are shown in Table S1. Samples (both healthy and diseased dogs) were collected and stored for up to 2 years prior to UEV isolation by SEC.

Nanoparticle tracking analysis confirmed the presence of particles with a size profile consistent with EVs (modal size @100 nm). TEM confirmed the presence of particles with size and “cup‐shaped” morphology expected of EVs (Figure 1). GO enrichment analysis of the proteomic data confirmed significant enrichment for proteins deriving from “exocytic vesicles” (false discovery rate = 0.00263, *P* = 1.48 × 10^−6^).

**FIGURE 1:**
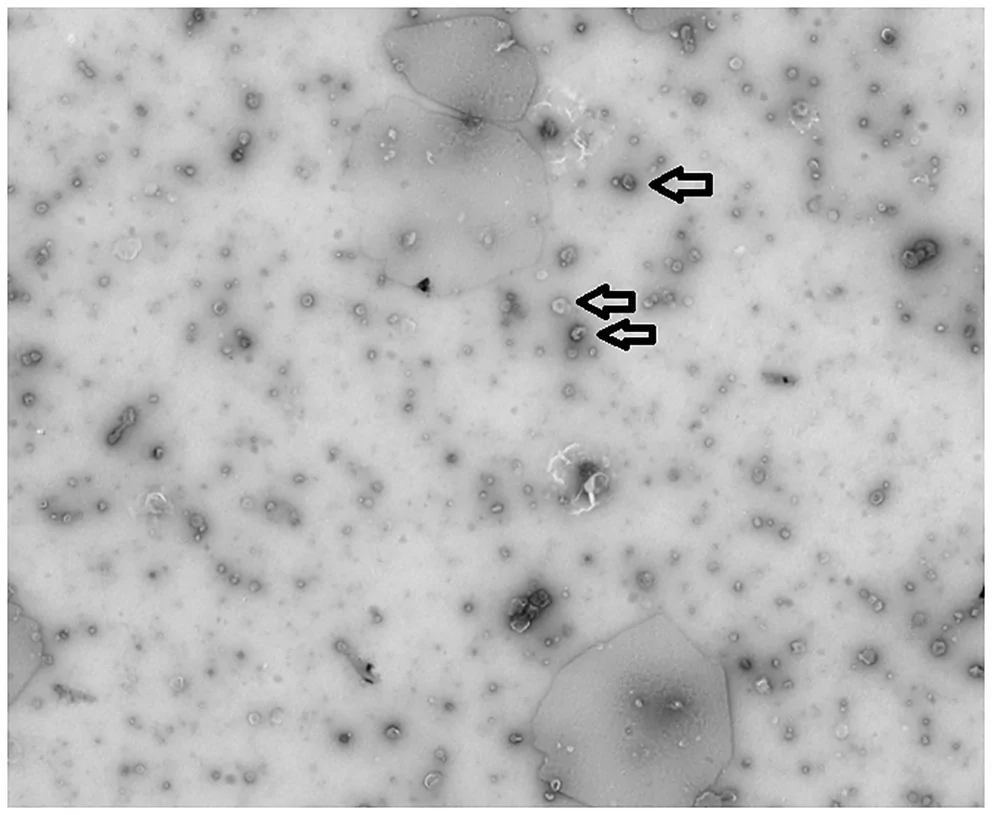
Transmission electron microscopy image of extracellular vesicles (EVs)‐enriched fraction obtained from the urine of a healthy dog. The image shows vesicles of ~100 to 200 nm diameter with the characteristic ‘cup‐shaped’ morphology expected of EVs (arrows).

The total creatinine content of samples was significantly different between groups (*P* = .03), with that from healthy dogs significantly higher than that of the samples from the hypercalcemic, hypercortisolism, and hypoadrenocorticism groups (*P* < .02 for all comparisons, Table S1). Similarly, the total number of EVs sent for proteomic analysis (ie, number of EVs in 500 μL of EV‐enriched fraction) was significantly different between groups (*P* = .03) and was higher in healthy dogs compared to the other groups (*P* < .02 for all comparisons, Table S1), likely reflecting the higher urine concentration and urine volume of the samples from the healthy dogs compared with the other groups. However, the UEV:creatinine ratio of samples (a surrogate marker of UEV excretion rate) was not significantly different between the four groups (Figure 2).

**FIGURE 2:**
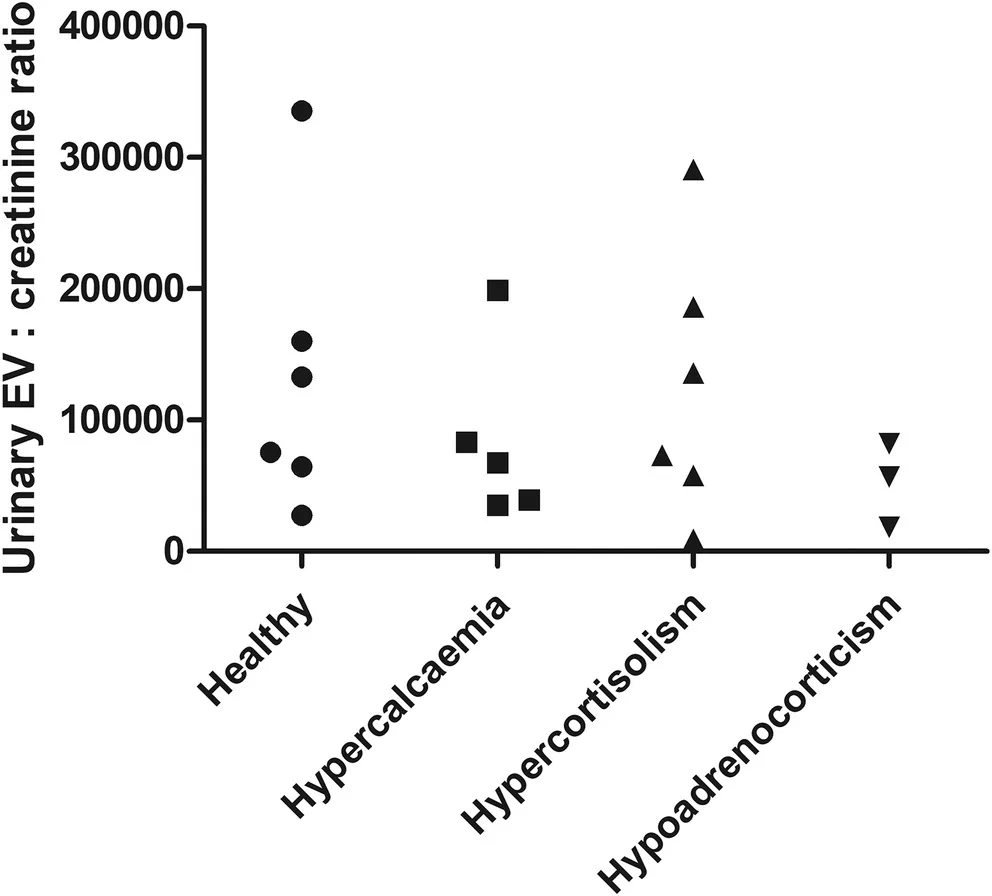
Scatter plot showing the urine extracellular vesicle concentration normalized to the urine creatinine concentration ratio of canine urine samples from healthy dogs, dogs with hypercortisolism, dogs with hypercalcemia secondary to primary hyperparathyroidism or anal sac adenocarcinoma, and dogs with hypoadrenocorticism.

Label‐free quantification (LFQ) data obtained for the proteins of interest are summarized in Table S1. Tumor susceptibility gene protein 101 (TSG101) and cluster of differentiation gene protein 9 (CD9, both protein markers of EVs) were detected in EV preparations from all healthy dogs; however, CD9 was only detected in EV preparations from one dog with hypercortisolism, and TSG101 was only detected in EV preparations from one dog with hypercalcemia and two dogs with hypoadrenocorticism. AQP2 was detected in EV preparations from all healthy dogs but none of the dogs in the other groups. NPT2A was detected in EV preparations from five out of six healthy dogs but only one dog with hypoadrenocorticism. ENaC and ROMK were not detected in any EV preparations, including those of healthy dogs. Unfortunately, comparison of LFQ for tubular transporter proteins of interest between the healthy and diseased groups was not possible due to the low number of dogs in which these proteins were detected in the diseased groups by LC–MS/MS.

AQP2 and NPT2A were both detected in samples from healthy dogs containing at least 1.7 × 10^9^ UEVs (Table S1). The number of EVs in the EV preparations was strongly positively correlated with the total creatinine content of the urine sample (*r*_s_ = .84, *P* < .001, Figure 3), indicating that the urine creatinine content of a sample could be used as a surrogate marker of the number of UEVs in the sample. AQP2 and NPT2A were both detected in samples from healthy dogs containing at least 24 μmol creatinine (Table S1).

**FIGURE 3:**
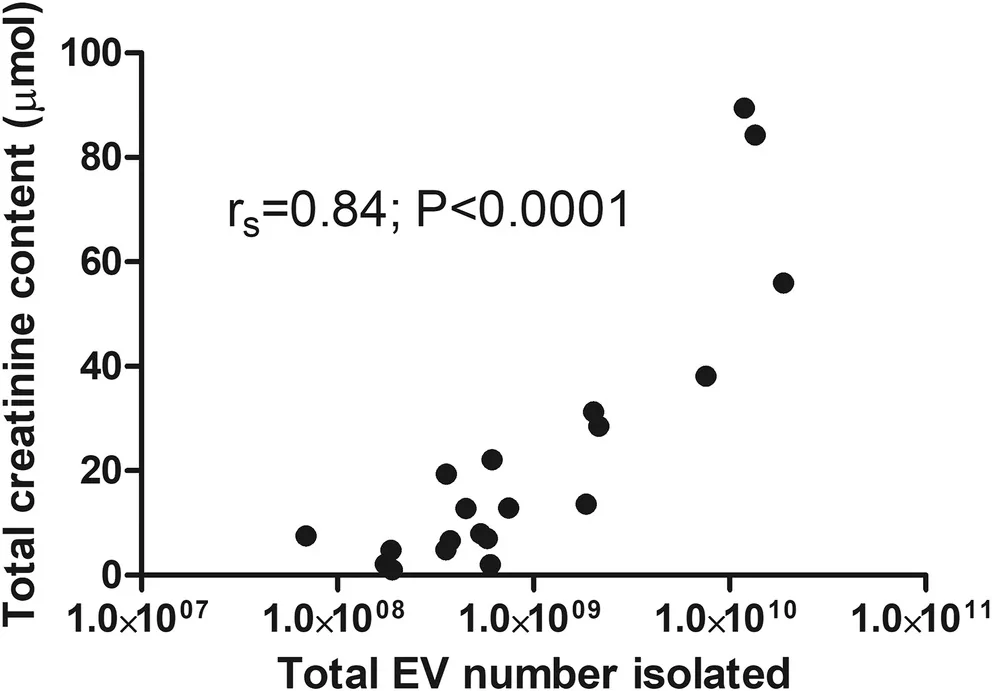
Correlation between the number of extracellular vesicles (EVs) in the EV‐enriched fraction and total creatinine content of each urine sample included in the study. Correlations were assessed using Spearman's correlation coefficient. There was a strong positive correlation between the total creatinine content of the sample and the total number of EVs in the EV‐enriched fraction.

## 4 DISCUSSION

In this study, we show proof of concept that renal tubular transporter proteins can be detected within UEVs of dogs. AQP2 and NPT2A were detected in UEVs from most healthy dogs, but they were detected in only one dog with primary hypoadrenocorticism and in no dogs with hypercortisolism or hypercalcemia. Given that in the present study, detection of AQP2 and NPT2A correlated with the number of UEVs, and UEV preparations from diseased dogs contained fewer UEVs, it is possible that the lack of detection of AQP2 and NPT2A reflects the lower number of UEVs in these samples, and thus the abundance of these proteins being below the limit of detection of the LC–MS/MS method. However, it should be noted that there was some overlap in the number of UEVs present in preparations for which these proteins were and were not detected, which could reflect variability in the abundance of these proteins on individual UEVs between individuals or inaccuracies in the measurement of UEV concentrations by NTA. The urine samples from the diseased groups were of lower volume and creatinine concentrations and thus contained lower total creatinine content than those obtained from the healthy group. The total creatinine content of urine samples was strongly positively correlated with the number of UEVs (Figure 3), as has been reported in humans.^2^ In our study, AQP2 and NPT2A were detected in samples from healthy dogs containing at least 1.7 × 10^9^ UEVs or 24 μmol creatinine; however, since there is a strong correlation between UEV number and total creatinine content of the samples, the urine creatinine concentration of the sample could be measured in advance of UEV isolation to determine whether sufficient UEVs are likely to be present in a sample of a given volume to permit proteomic analysis.

ENaC and ROMK were not detected in any EV preparations containing up to 1.6 × 10^10^ UEVs. We hypothesize that the abundance of these proteins in canine UEVs is low, and so the amount of these proteins was below the limit of detection of our LC–MS/MS method since both proteins have been detected in human UEVs.^14^ Further studies using urine samples containing higher numbers of canine UEVs would be required to establish the limit of detection of ENaC and ROMK using LC–MS/MS.

The UEV:creatinine ratio (EVCR) was calculated as a surrogate marker of the 24‐h UEV excretion rate since the urine protein:creatinine ratio can be used to estimate 24‐h protein excretion in dogs.^15^ In the present study, significant differences in EVCR were not observed between the healthy dogs and dogs in the diseased groups, which could suggest that the UEV excretion rate is not altered in hypercalcemia, hypercortisolism, or hypoadrenocorticism, although further studies, including larger numbers of samples, would be required to confirm these preliminary findings. The EV excretion rate correlates with the estimated glomerular filtration rate (GFR) in humans,^2^ and GFR is increased in dogs with hypercortisolism.^16^ Therefore, it could be hypothesized that urine EV excretion rate (EVCR) should be increased in canine hypercortisolism. Previous studies in humans have suggested that spot urine EV concentration normalized to urine creatinine concentration is not correlated with 24‐h urine EV excretion unless a further correction for 24‐h creatinine excretion rate is made, since this further correction will correct for differences in body muscle mass between individuals.^2^ Dogs with hypercortisolism will have reduced body muscle mass,^17^ which could reduce 24‐h urinary creatinine excretion; however, this should increase EVCR in hypercortisolism compared to healthy dogs, which was not observed in our study. Urinary 24‐h EV excretion rate is also correlated with total kidney volume (TKV) in humans,^2^ and it is possible that the changes in GFR that accompany hypercortisolism in dogs are not associated with changes in TKV and thus may not be associated with changes in 24‐h EV excretion rate. Further studies to investigate the changes in 24‐h EV excretion rates in dogs with hypercortisolism would be of interest to investigate this further.

Our study has a number of limitations. First, although we were able to show proof of concept that some renal tubular transporter proteins could be detected in UEVs isolated from small volumes of canine urine, many of the samples did not contain sufficient UEVs to detect the tubular transporter proteins of interest. This prevented us from comparing these tubular transporter proteins in different diseases, which are expected to alter the renal expression and abundance of these proteins. Therefore, we could not show proof of concept that UEVs are surrogate markers of the canine renal proteome, and further studies using larger volume urine samples would be required to demonstrate this. Second, EV protein markers (CD9 and TSG101) were not detected in EV preparations from most of the dogs in the diseased groups, which could suggest that these EV fractions contained very few EVs; however, significant numbers of particles with a size profile consistent with EVs were detected in all EV preparations by NTA. It is also possible that protein degradation occurred in the time between urine sampling and urine storage at −80°C since protein inhibitors were not added until the time of urine thawing and EV isolation. In future studies, it would be beneficial to add protease inhibitors to urine samples immediately after sampling to minimize degradation and potentially increase sensitivity for the detection of renal tubular transporter proteins in UEVs. Furthermore, the samples from the healthy dogs are likely to have been processed and stored at −80°C more quickly than the samples from the non‐healthy dogs, which may have allowed more protein degradation to occur in the samples from the non‐healthy groups. Finally, the samples from the healthy dogs were collected by free catch, whereas the samples from the diseased dogs were collected by cystocentesis, which may have caused differences in the calculated EVCR and the EV proteome since EVs from the urethra would not be included in the EV preparations obtained from the diseased dogs. However, the majority of UEVs appear to be derived from the kidney, and the proteome of UEVs isolated from voided samples is almost identical to that of UEVs isolated from samples taken directly from the kidney in the same patients^2^; thus, the contribution of urethral EVs to the total EV excretion rate and the EV proteome is considered likely to be small, although further comparisons of the EV proteome and number of EVs obtained from free‐catch and cystocentesis samples in dogs would be needed to confirm this hypothesis.

In conclusion, the renal tubular transporter proteins AQP2 and the sodium/phosphate co‐transporter NPT2A can be detected in UEVs obtained from canine small‐volume urine samples. Our data suggest that urine samples from healthy dogs containing at least 24 μmol of creatinine may be needed to isolate sufficient urine EVs to permit detection of AQP2 and NPT2A by LC–MS/MS.

## 5 CONFLICT OF INTEREST STATEMENT

The authors declare that they have no conflict of interest.

## Supporting information


Table S1.
